# The role of mast cells, interleukin-13 and transient receptor potential channels in a mouse model of chemical-induced airway hyperresponsiveness

**DOI:** 10.1186/2045-7022-3-S2-P31

**Published:** 2013-07-16

**Authors:** Fien Devos, Vanessa De Vooght, Benoit Nemery, Peter Hoet, Jeroen Vanoirbeek

**Affiliations:** 1 KU Leuven, Occupational, Environmental and Insurance Medicine, Leuven, Belgium

## Background

Occupational asthma is the most common work-related lung disease in industrialized countries. The mechanisms of occupational asthma caused by chemicals are still not completely understood. Therefore, we used a mouse model of chemical-induced asthma to examine the role of the neurogenic system as well as the role of IL-13 and mast cells by using different knock-out mice.

## Method

On days 1 and 8, wild type C57Bl/6 mice, IL-13, TRP (Transient Receptor Potential) A1, TRPV1 and mast cell deficient mice were dermally sensitized with 1% TDI (toluene-2.4-diisocyanate) or vehicle (acetone/olive oil) on both ears. On day 15, the mice received a single intranasal challenge with 0.1% TDI or vehicle. In a second experiment TDI or vehicle sensitized wild type C57Bl/6 mice received an intraperitoneal injection of the NK1R antagonist RP67580 (1µg/µl) prior to the challenge. Airway reactivity to methacholine, lung inflammation, lymphocyte subpopulations in the draining auricular lymph nodes and total serum IgE were assessed 24h after the challenge.

## Results

IL-13, TRPV1, TRPA1 and mast cell deficient mice showed a significant lower airway hyperreactivity compared to wild type mice, 24h after TDI challenge, without any sign of lung inflammation. Treatment with the NK1R antagonist also resulted in a significant decrease in airway hyperreactivity. In the auricular lymph nodes T-helper cells, T-cytotoxic cells and B-cells were significantly lower in mast cell deficient and IL-13 deficient mice, compared to wild type mice.

## Conclusion

These results indicate the importance of IL-13, TRPA1 and TRPV1 channels and mast cells in the development of immune-mediated bronchial hyperreactivity.

